# Horizontally Distributed Inference of Deep Neural Networks for AI-Enabled IoT

**DOI:** 10.3390/s23041911

**Published:** 2023-02-08

**Authors:** Ivan Rodriguez-Conde, Celso Campos, Florentino Fdez-Riverola

**Affiliations:** 1 Department of Computer Science, University of Arkansas at Little Rock, 2801 South University Avenue, Little Rock, AR 72204, USA; 2 Department of Computer Science, ESEI—Escuela Superior de Ingeniería Informática, Universidade de Vigo, 32004 Ourense, Spain; 3 CINBIO, Department of Computer Science, ESEI—Escuela Superior de Ingeniería Informática, Universidade de Vigo, 32004 Ourense, Spain; 4 SING Research Group, Galicia Sur Health Research Institute (IIS Galicia Sur), SERGAS-UVIGO, 36213 Vigo, Spain

**Keywords:** IoT, collaborative inference, deep neural networks, distributed computing, DNN splitting, task offloading, mobile edge computing

## Abstract

Motivated by the pervasiveness of artificial intelligence (AI) and the Internet of Things (IoT) in the current “smart everything” scenario, this article provides a comprehensive overview of the most recent research at the intersection of both domains, focusing on the design and development of specific mechanisms for enabling a collaborative inference across edge devices towards the in situ execution of highly complex state-of-the-art deep neural networks (DNNs), despite the resource-constrained nature of such infrastructures. In particular, the review discusses the most salient approaches conceived along those lines, elaborating on the specificities of the partitioning schemes and the parallelism paradigms explored, providing an organized and schematic discussion of the underlying workflows and associated communication patterns, as well as the architectural aspects of the DNNs that have driven the design of such techniques, while also highlighting both the primary challenges encountered at the design and operational levels and the specific adjustments or enhancements explored in response to them.

## 1. Introduction

As a result of a steady synergy still currently in place, IoT and AI have almost simultaneously experienced outstanding progress in the past two decades, leading to the so-called AI-enabled IoT and thus achieving the vision of a pervasive intelligence [1]. Methods and technologies developed under the IoT paradigm facilitate the connection of the different devices that comprise such intelligent environments and the exchange of data between them [2], enabling the creation and proper exploitation of new network architectures consisting of connected ambient sensing instruments and resource-constrained energy-efficient end devices (i.e., embedded devices and mobile devices), commonly referred to as user equipment (UE) in the related literature. Efforts in this regard have been primarily aimed at designing and deploying faster and more efficient network infrastructures, as well as developing more accurate sensing platforms. This has dramatically increased the capability of those systems to sense data from the physical world, thus enabling the collection and storage of large volumes of data and consequently supporting increasingly sophisticated AI techniques—from traditional machine learning (ML) methods to more recent deep learning (DL) approaches—ultimately creating enormous opportunities for a “smart” life.

Deep neural networks (DNNs) have driven the evolution and subsequent consolidation of “intelligent” computing systems among the general public beyond research forums, proving their undeniable power and achieving great success in a number of application domains, such as smart transportation [3], smart farming [4], smart manufacturing [5], and smart healthcare [6]. Exploiting the sensors embedded in these systems has enabled massive data collection, nurturing deep learning algorithms and thus contributing to achieving new levels of accuracy in the delivered results. However, this accuracy has come at the cost of an increase in computational and memory resource consumption [7], both in terms of training the networks as well as making inferences. Moreover, in the particular case of inference, it entails more than just numerous complex computations and memory expansion of the processed DNN model, which are already relevant challenges for IoT devices; for most of the use cases found in the typical application domains of IoT systems mentioned above, time is a critical factor, and real-time prediction performance is expected from them.

Even though today there are hardware solutions specifically for AI acceleration in embedded and mobile devices that could be considered, to some extent, as a possible answer to the above needs, they have been shown to be insufficient to efficiently address the execution of today’s more sophisticated DL models. As a result, GPU cluster-powered cloud-based configurations are still the standard DL research-support infrastructure used today. Nonetheless, the reliance on remote data centers—i.e., geographically located far away from the users or the data sources—for DNN execution may incur prohibitive latency delays, thereby failing to meet the minimum latency goal pursued. In this context, new computing paradigms, namely Mobile Cloud Computing (MCC) and Mobile Edge Computing (MEC) [8], have emerged over the last few years as alternatives to classic cloud computing (CC). These have also impacted the AI domain, enabling the progressive abandonment of the latter’s fully cloud-delegated processing model towards a vertically distributed computation across the EU–edge–cloud continuum, bringing part of the DNN inference to computing tiers closer to end users, and thereby resulting in the so-called collaborative inference [9].

Specifically, this collaborative intelligence has been materialized at a practical level, essentially in the form of a pipeline for the execution of DNNs on multiple entities distributed across the different levels of computation considered, although subjected to the structural properties of the models utilized. In this sense, although it is an approach that succeeds in generating segments of reduced size and complexity through the partitioning of deep networks, allowing devices with limited capabilities to take on some of the load and offload the heavier subtasks to nodes at higher layers of the hierarchy [10,11], its effectiveness is to some extent undermined or constrained by the challenging issues that remain, such as the significant distance between nodes, the adoption of the layer as the minimum partitioning unit, and the inter-layer data dependency inherent to DNN models. Hence, such an approach results in end-to-end latency numbers that, dominated by data communication times, fail to achieve the real-time goal [9]; it also allows the generation of partitions that, even in their minimum expression, may result in a memory footprint and computational load excessive for IoT devices; and, last but not least, it further penalizes the overall co-inference performance by preventing the processing of DNN partitions to be handled concurrently.

Given these limitations, recent studies have explored other alternatives of computation at the edge, introducing novel methods and strategies aimed at better leveraging the distributed resources within the same computing tier towards a future vision of extremely interoperable and flexible AI-capable IoT systems. Specifically, in this context, such research has pursued the design of cooperative inference mechanisms [12] that, unlike those mentioned above, demonstrate the capability to speed up the execution of DL tasks by partitioning the workload and distributing the resulting DNN segments horizontally across the devices within an edge cluster, whether this is referred to as a mini-cloud (i.e., a cluster of computers within the same LAN [13]), a micro-cloud (i.e., an infrastructure-independent and easily portable assembly of small computers [14]), an ad hoc cloudlet [10] (a cluster consisting of mobile devices interconnected via short-range radio communication technologies), or a fog network [15] (perhaps the most representative term, referring to an architecture consisting of end-user clients or near-user edge devices that can alternatively cooperate and support machine-to-machine-based service provisioning in a distributed manner).

In such a context, determining how to efficiently partition, distribute, and schedule DNN inference within such an environment, considering the significant heterogeneity of devices regarding their capability, including edge servers equipped with GPUs, low-power single-board computers such as the Raspberry Pi, and smartphones with multi-purpose Systems of a Chip (SoCs), and the dynamic network conditions, continues to pose major challenges that make the edge-based efficient AI service provisioning an open and still relevant research problem. For this reason, in the present work, we conduct an in-depth study of the most relevant aspects of this edge intelligence (EI). This research, far from being an exhaustive or systematic review of the related literature, aims to be a comprehensive and gentle introductory guide to those techniques and methods that have proven to be highly successful in exploiting the aggregated computational and memory resources of in-cluster IoT nodes to address highly complex DNN tasks in a timely manner and to ultimately deliver a desirable quality of service (QoS) despite the acute resource limitations of the interconnected devices.

It should be noted, however, that, while computation offloading at the edge and the distribution and deployment of deep learning solutions on such computing environments are still emerging topics that have gained momentum over the past five years, both have already given rise to a vast corpus of scientific articles, leading to a fairly important number of surveys, as illustrated in Table 1. Specifically, we found sixteen papers [11,12,16,17,18,19,20,21,22,23,24,25,26,27,28,29] focused on EI that provide an extensive overview of the current state of the art in the topic space. They guide the reader through a comprehensive collection of methods and technologies designed to better leverage edge infrastructures for DNN training [11,12,16,17,21,22,29] but primarily for the execution of such DL models [11,12,16,17,19,20,21,22,23,24,25,26,27,29].

Regarding inference, an extended trend is particularly noticeable among the authors on providing an overview of architectures and workflows for enabling DNN processing at the edge, giving particular attention to (i) techniques that make DL models applicable for direct deployment and local execution on resource edge devices by creating lightweight architectures from scratch [11,12,16,23], i.e., naturally suitable for edge environments, or by adjusting existing DNNs to reduce their complexity and size [12,16,17,21,22,23]; and (ii) strategies pursuing the realization of an offloading-based collaborative inference across multiples devices located either at the same tier or in different computing levels [20,22,23,24,25]. Moreover, most authors extend (i) and (ii) by introducing core concepts [18,24] and providing extensive background on other EI-specific matters such as (i) the most representative application scenarios [12,18,21,27], (ii) the software [21,29] and hardware infrastructure [16,20] for facilitating EI, and (iii) the most relevant challenges that need to be faced for its realization, i.e., model partitioning [11,17,19,22], communication [11,26], edge coordination [20,27], and more AI-related challenges [20].

Although the studies cited cover a wide range of relevant topics at the intersection of edge computing and AI, and may serve as a good starting point for gaining an understanding of the distributed execution of DL algorithmic solutions and for establishing a foundation for the knowledge that will be progressively solidified throughout the rest of this paper, they go beyond the scope of the studies cited, providing a higher-level overview of edge-based AI and, as a result, differing in the thematic core and the level of detail embraced. Overall, the narrative style used adeptly guides the reader through the various approaches conceived, introducing the related concepts pertinent in each case without delving too deeply, omitting a significant number of the underlying design considerations, and thus falling short when it comes to discussing the techniques and methods presented. To fill this gap, our work supplements the existing body of literature and provides a comprehensive and in-depth review of the most salient studies published that have led to the emergence of cooperative intelligence solutions in IoT environments, discussing the specific approaches and strategies conceived for partitioning and parallelizing DNNs, providing an in-depth treatment of the decision-making process required, and, finally, giving details on both the different challenges or issues that have emerged in this domain and the specific solutions conceived in response. 

This analysis relies on a corpus that was meticulously and systematically chosen through a comprehensive and extensive process of incremental discovery, where the concerns of interest outlined in the previous paragraph, as well as the nature of the supporting infrastructure and the operational constraints adopted, i.e., tagging inference-related computations at the same tier within the underlying architecture, guided the search and filtering of the various sources finally used as a reference. Notably, keywords were carefully selected to be in line with the objectives of the research, aiming primarily to obtain relevant articles while ensuring that none were left out of the search results. Accordingly, employing Google Scholar as the main search engine, the initial screening of the literature was carried out using the following terms: “collaborative intelligence”, “collaborative inference”, “distributed inference”, “split computing”, “DNN partitioning”, “DNN splitting”, “DNN parallelism”, “in-cluster co-inference”, “DNN co-inference scheme”, “DNN offloading”, “joint model split”, “model partitioning”, “model slicing”, “DNN task distribution”, “distributed deep neural networks”, “split machine learning design”, and “hybrid DNN computation”. The list of 132 entries obtained from the first cursory reading of the abstract and the conclusions of each paper was then refined by an in-depth reading and thorough analysis of these research efforts of potential interest, assessing their quality and excluding those considered outside the domain of study, either due to having no place in the IoT paradigm—belonging instead to other closely related fields, such as the already mentioned MCC, MEC, or CC—or because, like [30,31,32], despite being under the IoT umbrella and being also related to the distributed computation model, they were oriented instead to locally distributed solutions relying on modern SoCs for parallelization.

The methodology for examining and selecting the literature just outlined resulted in a body of work that, albeit small, made it possible to clearly outline the most relevant aspects of cooperative intelligence in IoT contexts. More specifically, all these aspects are developed throughout this document according to the following structure. The second section puts the study in context by briefly presenting some of the most relevant milestones in the evolution of both AI and computing platforms towards deep learning at the edge. Specifically, and with regard to this last point, Section 3 examines the various research efforts on the distribution of DL workloads within IoT clusters, establishing a taxonomy of the primary parallelism strategies and partitioning schemes proposed, and elaborating on their most pertinent aspects at the practical level, the pillars of the decision making required for their proper configuration, as well as the specific challenges that arise when addressing the setup and the effective exploitation of such mechanisms. Section 4 shares the observations drawn from the state of the art and highlights the still open research challenges to be addressed in future work, while Section 5 brings the survey to a close by presenting the conclusions.

## 2. Background: Towards Deep Learning at the Edge

The synergy between the IoT and AI has been, without a doubt, a driving force behind the remarkable growth experienced by both realms almost simultaneously over the past two decades, leading to the so-called “smart” environments and “smart” things. Such an interaction, which is still in place today, although accelerated by the recent thrust of deep learning (DL), has been, to a large extent, a natural evolution of methods and technologies strictly conceived under the umbrella of the IoT paradigm toward the creation and proper exploitation of network-based architectures consisting of interconnected ambient sensing instruments and resource-constrained yet energy-efficient devices, e.g., embedded and mobile devices, with the capability of gathering and exchanging data. In that sense, research and engineering efforts that were originally conceived for the design and deployment of faster and more efficient network infrastructures, as well as the development of more accurate sensing platforms and capable computing hardware, have also enabled the collection and storage of large volumes of data, thereby supporting increasingly sophisticated AI techniques, from traditional ML to DL.

Advances in DNNs envisioned in AI fields as diverse as computer vision, natural language processing, and automated speech recognition, have shown an unprecedented ability to learn abstract representations and extract high-level features from sensed data, delivering, as a result, highly accurate and robust predictions [33,34] from the input provided. A clear example of that is convolutional neural networks (CNNs), which have emerged over the last decade as the state of the art for vision tasks, constituting an information extraction method superior to more traditional approaches [35] and capable of generating higher-resolution semantically richer representations from imaging data. However, this progress, not only regarding accuracy but also in terms of the precision of the output produced, has been accompanied by an increase in computational and memory needs [7], especially for the training phase, but also for the exploitation of the various resulting models during the inference process. DNNs have evolved towards more profound and complex network designs, often comprising a massive collection of parameters, in many cases numbering in the millions, e.g., VGG-16 [36] has 138 M parameters and AlexNet [35] has 61 M parameters. This has had a direct effect on training, which iteratively refines a large number of parameters over multiple periods, resulting in higher memory and computational costs, and on inference, which is also computationally expensive, due not only to a large number of parameters but also to the potentially high dimensionality of the input data (for instance, a high-resolution image), the millions of computations that must be performed on them, and the very complexity of some of the operations involved, such as multiply-accumulate operations [37].

Modern smart IoT devices are equipped with powerful integrated chips that can be utilized for in situ data processing. AI-specific capabilities of novel hardware solutions—including embedded devices and accessories for accelerating ML tasks, such as application-specific integrated circuits (ASICs) [38,39], GPUs [40,41], and FPGAs [42,43]—have emerged as the infrastructure cornerstone of the recent AI trend known as on-device ML, underpinning an intense and still ongoing research activity that has eventually led to a plethora of methods and techniques that enable the deployment and execution of DL solutions directly aimed at UEs. More specifically, in that respect, scientists and experienced practitioners have explored not only how to better leverage such AI-optimized highly efficient devices, but also how to conceive more compact and lightweight DNNs, which is in turn pursued through the compression and simplification of existing networks, e.g., quantization [44], sparsification [45,46], and pruning [44], as well as the creation from the ground up of more efficient architectures [47,48]. Both lines of research have proven to be beneficial, reducing the memory footprint of DNNs and accelerating the execution of the underlying computations [49]. 

In most cases, however, that performance upgrade has been achieved at the expense of high energy consumption, totally at odds with the very nature of IoT devices commonly deployed in the wild and, as such, with no access to a long-standing and steady power supply. Moreover, and more importantly, both the accuracy delivered and the latency reduction achieved as a result of the research undertaken along those lines have been shown to be still insufficient to implement intelligent systems on IoT computing platforms. Accuracy and, especially, latency make it difficult for such resource-constrained devices to provide users with a more fluid and better-tailored experience [50] without compromising data integrity, and are reported to be far from the optimum required to support some latency-sensitive and safety-critical applications such as augmented reality [51] and cooperative autonomous driving [52], which have been traditionally associated with, and actually emerged under, the umbrella of this type of computing entity.

Thus, although the solutions outlined above, conceived following a mobile computing model, have become leading facilitators for large-scale data collection, they still fall short when it comes to dealing efficiently with the compute-intensive tasks in the DL workflow. In this regard, cloud platforms, consisting of highly scalable configurations backed by virtually unlimited computational and memory resources, remain the standard infrastructure for accelerating DL tasks today. Particularly in this respect, CC has played a crucial role at the operational level in DL, not only enabling DNN architectures to be trained in a relatively reasonable amount of time and the resulting highly complex models to be executed in a timely manner [53,54], but also hosting and making such models available as remotely accessible services, making them exploitable by other online devices regardless of their system resources settings or current burden. In that context, one of the traditional methods used to solve the challenges posed by DL on IoT and overcome the limitations of the commonly exploited embedded and mobile devices has been to transfer the computation-intensive tasks and service data from UEs to the more powerful remote cloud infrastructure [55,56].

That cloud-based DNN computation was a prominent trend during the past decades, dramatically pushing forward DL-specific research through a computation offloading strategy conceived as a cloud-only scheme. In such an offloading strategy, the cloud takes on the entire operating load acting as a surrogate, relegating UEs to a secondary role in which their only responsibilities are (i) capturing the user interaction or sensing the environment, (ii) making a request to the server for each and every DL job, and, finally, (iii) presenting or exploiting the received results [57]. This UE–cloud dialogue typically entails the transmission of a substantial amount of data via a wireless medium between geographically distant nodes. This necessary vertical communication poses major issues in terms of response time [58], availability [59], robustness [60], and security [61], constituting these especially significant hurdles in IoT contexts. In that sense, incorporating DNN capabilities into IoT devices further increases their dependency on the availability of data centers with high quality of service and network resources while, at the same time, the rapidly rising number of devices and quantity of data traffic in the current IoT era pose significant burdens on capacity-limited networking infrastructures and an unmanageable service latency, hindering the possibility of meeting the delay-sensitive and context-aware service requirements of a great deal of the IoT applications listed in the previous section by merely leveraging CC.

Such a classic vision of the cloud, similar to the more traditional client–server model consisting of a thin client requiring services from a powerful server, has given way in recent years to new computing paradigms that have progressively sought to alleviate the workload on the cloud, and bring more capable server entities closer to either end users or the data collection infrastructure deployed, i.e., the MCC and MEC paradigms. In particular, MCC, unlike the more traditional CC paradigm, represents a computational augmentation approach that advocates for the partial offload of UE’s burden on the cloud as a method for reducing the processing and storage needs of terminal devices, as well as increasing their battery life. With specific regard to DL tasks, such an approach and its underlying philosophy have already reflected on how DNNs are executed in such constrained contexts, constituting the groundwork for processing the exploited models through a cooperative intelligence strategy. This strategy, realized in the form of a collaborative inference—also referred to in the related literature as co-inference—has emerged in the past few years as a natural evolution of the on-device and cloud-centric ML approaches mentioned above, pursuing the joint utilization of the cloud and the UEs through a vertical distribution of the inference, partitioning the DNN used, and then deploying the resulting segments on the computing tiers involved.

Although it traditionally refers to a flexible server platform with vast computing power, high memory resources, and a stable and sustained power supply [62], the cloud term is expanded in the MCC paradigm beyond the model inherited from CC, including any remotely accessible infrastructure within the boundaries of the system regardless of the type of hardware configuration used and the number and combination of nodes that comprise it [63]. That revised formulation of the concept encompasses solutions that, while representing a slight step back in terms of power, bring the cloud closer to user-level devices, thus mitigating the limitations of classic cloud systems, accessible only over wide area networks and blurring the lines between MCC and MEC. In that sense, although MEC systems have recently emerged as a complement to CC for performing computations on the network edge [8], creating a great buzz in both academia and industry due to their promise of maintaining closer proximity to the user, in their simplest form they repeat a formula similar to the one described for MCC, relying on cloudlets [64]—described, in the authors’ own words, as a “data centre in a box”—to also enable “server-class” applications. Cloudlets, while a server infrastructure less capable than the elastic cloud, constitute a more cost-effective alternative. They feature physical and network specifics that facilitate their deployment in strategic locations, e.g., public installations and private servers, operating as a server entity of terminal devices, supercharging them with nearby extra processing power and storage space, and even serving in some scenarios as an intermediate computing tier between the latter and the cloud.

Therefore, MCC and cloudlet-based MEC can be viewed as being operationally very similar approaches, sharing, as such, the previously noted co-inference realization. Along these lines, AI researchers have already proposed a good number of techniques [9] that have proven to be successful at distributing the execution of a given DNN across the UE–edge–cloud continuum in the form of a pipeline, partitioning the DNN structure in the origin and offloading part of it—typically partitions with the highest complexity—into the more capable computing tiers. Such a strategy socializes the computation costs, enables scaling the inference on DNNs vertically through the collaboration of nodes located at different computing levels, and facilitates exploiting the hardware capabilities in the support infrastructure in a more rational manner, particularly in MEC systems where, in addition to UEs, edge devices can be leveraged to alleviate and even, in some cases, eliminate the dependency on the cloud to ultimately reduce the end-to-end latency and thus improve the quality of service provided.

That said, pipeline processing, having been recognized as a promising way to augment resource-constrained devices and, at the same time, reduce the computational load and memory consumption per entity involved, has been shown to be a solution that falls short when it comes to actually getting such computing entities engaged in the co-inference process, achieving a reduction in the indicated demands commonly unsatisfactory to accommodate the execution of DNNs, and a decrease in the level of latency not substantial enough to ensure the performance required for time-critical applications [9]. Specifically, while MCC still suffers from significant communication latency and limited backhaul bandwidth, MEC has been able to mitigate such transmission costs by primarily pushing computing tasks to small, nearby cloudlets, thereby avoiding time- and energy-intensive long-distance transmission [65]. Moreover, with regard purely to the computations involved in DNN inference, both MCC and MEC systems rely on the aforementioned vertically distributed scheme, which, overall, resorts to inter-layer partitioning methods that (i) result in partitions that might lead to a memory footprint and computational burden not low enough for IoT devices, and, more importantly, (ii) are subject to data dependency between partitions, which prevents actual parallelism and thus undermines concurrent exploitation of multiple devices. Finally, it should also be noted that privacy remains a challenge: even though MEC inherently benefits privacy by keeping local data at the edge of the network, there is often a need to exchange some data between edge devices and the cloud.

Therefore, while today’s IoT systems can recognize and monitor their surroundings, collecting data in the form of still images, video, or data streams, and modern advanced DL solutions are able to process those sensed data and derive high-level knowledge, enabling intelligent computational systems to extract context-specific knowledge and, as a result, provide more effective assistance, there are still important privacy, latency, and performance issues in the current “smart everything” scenario, tightly coupled to highly capable remote server entities located either on the cloud or at the edge. Such challenges not only remain pertinent but have particularly piqued the interest of the AI and IoT communities in recent years, paradoxically due to the relevance that mobile-based intelligent systems have acquired in today’s technological landscape beyond research forums.

## 3. In Situ Distributed Intelligence

The necessary shift towards a more rational close-to-data-source computing pattern has led to the consolidation of computing paradigms far removed from the previously mentioned server-centric approach, leading to distributed DL processing patterns within AI, i.e., collaborative inference, enabling a genuine workload sharing among the UE–edge–cloud continuum, and thus partially alleviating the burden of the edge and the cloud through effective exploitation of the resources available on the terminal devices. Seminal studies along these lines, e.g., Neurosurgeon [66], IONN [67], DADS [68], and QDMP [69], while still far from being sufficient approaches to achieving the objective stated above, have been instrumental in laying the foundations of DNN task partitioning and offloading, identifying key concepts and resulting in techniques that have had an organic translation to the edge, fog, and local environments, and paving the way for an in situ distributed intelligence model in those contexts.

With regard to the resulting techniques, following the topic identified by the research referred to in the previous paragraph, there is already an important body of work that has contributed to the widespread adoption of DL techniques in application domains where lightweight sensing solutions are explored and leveraged for building intelligent IoT systems. The information presented in Table 2 provides context and presents a chronological overview of the various research efforts that have driven the emergence of novel co-inference schemes articulated on the cooperation among the computing entities scattered all over the exploited hardware infrastructure to overcome their individual resource limitations, and thus support computationally demanding and memory-intensive DL workloads. As we can see, over the last five years, a fairly large number of authors have worked towards an in-cluster collaborative execution of the DNN models, designing partitioning and task allocation strategies that, to a greater or lesser extent, have been shown to be successful at distributing the required computations across the nodes within the same computing tier, keeping that processing near to the service requester.

In total, Table 2 collects details on twenty-two pieces of research reporting solutions for both the decision making and runtime required for the horizontally distributed execution of DNNs in IoT environments. The table provides an at-a-glance view of the major milestones within the context of interest, presenting the most significant aspects and issues addressed in the various studies analyzed, thereby establishing the basis for the discussion that will be developed throughout the rest of the paper. Specifically, the table in question not only provides background-related information such as the year of publication or the design objectives pursued in each case, it also constitutes a shallow taxonomy of the multiple approaches conceived, categorizing the latter according to its very nature, the application areas targeted, and the specific tasks supported. This also details the most relevant defining factors both at the architecture (as pertaining to infrastructure) and the operational (workflow-specific) levels.

In particular, regarding applications and tasks, the research conducted in this vein is still in its infancy and, in general, has not been specifically contextualized inside any particular scenario or application, with the exception of three studies. We mainly observe, therefore, general-purpose efforts conforming to a body of research that, far from being domain-specific, explores wide-scope approaches when it comes to the application targeted in order to meet the requirements that the deployment and execution of state-of-the-art DL tasks inevitably impose on resource-constrained physical devices, even when distributed among multiple devices. Practically, the discussion of potential use cases is put on the back burner to the extent that, even in those studies that make explicit reference to any of the use cases, the authors do so only to enrich the narrative and better illustrate the framework or purpose of the technique analyzed.

Regarding these tasks relying on artificial visual perception, image classification and object detection methods have emerged as two of the most important enabling factors of this “nearby” intelligence, providing a better understanding of the environment and the entities in it, and constituting a fundamental pillar for resolving relevant vision tasks such as human activity recognition in killer AI-backed IoT applications such as surveillance and analytics on data from wearable sensors. Moreover, beyond the significance of the tasks for evaluating and assessing the approaches developed, their selection has been an almost self-imposed obligation rather than a decision-making exercise, being fundamentally marked by the network architectures and, more specifically, by the types of layers adopted as the object of interest. Their analysis stands out in the scientific corpus examined almost as an imperative at the early stages of the research, allowing the authors to gain the knowledge necessary to properly marry the characteristics of the DNN models used and the specificities of the different elements or components that make up the supporting infrastructure.

Notably, CNNs, i.e., DNNs with at least one convolutional layer within their inner structure, have been reported as the sole architecture under consideration in the studies reviewed. First introduced by LeCun et al. [7] in 1989, CNNs have emerged as a major driver of progress in image analysis and computer vision over the past decade, producing accurate results at the cutting edge of the field. Despite their complexity, they have become one of the most paradigmatic DL models due to their structural characteristics, inspiring and spawning a multitude of research efforts that have proven to be successful at parallelizing the operations involved not only in the training process, but also in the execution of such models on MCC and MEC systems. In this regard, the studies presented in Table 2, heirs to those efforts, have explored and provided evidence of the pertinence of the CNN architecture to distributed computation techniques. The former not only poses a pipeline-like topology at the macro-architecture level, but also consists of highly parallelizable operations at the intra-layer, thus making it possible to apply methods, such as DNN partitioning and the allocation of the resulting subtasks, on the operations in the pursuit of a more efficient inference process.

While the evolution of the computing paradigms, specifically from CC to hybrid edge-cloud MCC and MEC computing paradigms, has been spurred by the emergence of latency-sensitive applications, both latency cost reduction and accuracy preservation have emerged as a major focus of interest among computer vision experts and, as such, have guided the search for solutions of this nature within the specific context of the present review. Quality of experience stays at the top of the roadmap, with its realization being pursued through the improvement in parameters primarily pertaining to the performance and, to a lesser extent, the cost of the distributed AI solutions proposed. In particular, performance refers to the efficiency of the systems or methods conceived, and the quality of the predictions produced by the DNN models used, whereas cost reflects the demand for resources entailed by the tasks involved.

Regarding performance, although the horizontally distributed synergistic computation scenario differs at the operational and infrastructure level from CC, MCC, and the more classic vertically distributed MEC contexts, most of the studies surveyed have focused on typical requirements of user-centric intelligent systems, seeking to develop methods and techniques for streamlining and accelerating the execution of DNNs to ultimately achieve a performance as close to real time as possible while maximizing accuracy or, at least, mitigating the accuracy loss so as to obtain an adequate latency–accuracy trade-off as a result. End-to-end latency, which refers to the time required to provide the user with a response to a requested DNN task, stands out as the most considered performance metric. As we shall see later, the process of addressing a DNN task in a distributed context extends far beyond computation, strictly speaking, to encompass the communication of intermediate results between the devices involved as well as the decision making to distribute and parallelize the workload.

On another front, cost objectives reflect the demand for hardware-dependent resources, referring to factors that arise from the execution of heavy DL tasks on devices with limited computation, memory, and energy budgets, which are themselves exacerbated in distributed IoT environments due to their need for pervasiveness, context awareness, and shared processing close to end users. Particularly in this respect, the data in Table 2 evidence an existing concern among the authors to reduce the memory footprint and the energy consumption to accommodate the utilized DNN models to such constrained computing entities. 

Memory constraints in UEs and edge devices have been shown to stymie DNN execution in cloud-only and pipelined computing schemes. Inference, whether performed as an end-to-end non-divisible process in cloud platforms or distributed across multiple computing entities in MCC or MEC environments, embraces individual layers as the minimum structural unit of DNN architectures, and, consequently, even in a best-case scenario, i.e., a single layer executed on a single device, it may still be too demanding for some of the specialized hardware architectures that, as mentioned in the previous section, have been designed for accelerating AI computations. Additionally, when it comes to energy consumption, although not unique to IoT solutions, it is indeed especially problematic in such contexts [92]. In contrast to edge and cloud platforms, which are typically powered by a stable energy supply and mainly aimed at providing support to not-so-capable pieces of equipment—subordinating, therefore, energy consumption to computing power—IoT systems are conceived as pervasive ecosystems comprising small-form-factor devices embedded in the environment and, because of that, do not commonly have access to a steady power source, being powered by relatively short-life batteries instead.

To overcome the limitations described, the explored approaches have followed the line laid down by the research efforts on collaborative inference referred to at the beginning of the section, taking steps towards a finer-grained partitioning and actual parallelism of the required computations. Except for a few cases that have focused on a specific aspect or have been circumscribed to a specific type of method, the authors have approached both lines of work in a comprehensive manner, resulting in frameworks that, designed as a distributed software infrastructure, integrate the algorithms necessary to break down DL requests into less costly DNN partitions that are transferred to and run on different computing entities belonging to the same computing tier while being located either physically close to each other or, on the contrary, more geographically scattered. To be even more precise, these frameworks have a three-fold purpose, typically serving at the same time as (i) a decision-making engine necessary for configuring and synthesizing either a partitioning plan or even a more sophisticated co-inference scheme, i.e., an execution plan that defines how to coordinate the devices involved properly; (ii) a scheduler, governing resource allocation and the distribution of DNN partitions; and (iii) a production environment or runtime, enabling the latter to be executed in a distributed yet cooperative way by exploiting the capabilities of the supporting hardware infrastructure.

Even though configuration—“partitioning” (PART) in Table 2 -, distribution—“task assignment” (TSK ASSG) in the same table -, and inference have different requirements and involve different tasks, they are all connected in some way and happen in a certain order, being considered part of the same workflow. It is for this reason that co-inference frameworks, at the operational level, are typically modeled as a pipeline consisting of two distinct stages that ultimately lead to the practical implementation of the co-inference scheme. Far from common task grouping factors—usually, the scope of the task or its object of interest—their breakdown into distinct stages follows time-based criteria and has nothing to do with the specific nature or purpose of each task. The first stage, represented in Table 2 by the “Offline setup” column, encompasses tasks commonly executed one time only at the initial configuration of the framework, while the second stage, referred to as “Runtime”, involves tasks executed once the system is deployed, typically upon request.

Taking a deeper look at the data collected in both columns, the analysis reveals a dominant scheme or baseline that, with minor exceptions, has stayed relatively consistent across the multiple frameworks devised to date since the publication of MoDNN in 2017 [70]. The decision-making engine stands as the primary responder in the initial system configuration stage, setting both the parameters that govern how to perform the partitioning of the DNN used and, to a lesser extent, the target nodes of the resulting partitions. Task assignment is, however, a job usually performed at runtime in response to each incoming DNN query, and, as we will see below, it may vary depending on the underlying communications architecture. The framework runtime, which is instantiated on each node that integrates the hardware infrastructure, is responsible for partition allocation and cooperative inference. Each computing entity is responsible for performing the computations required for the execution of a given partition, transmitting, once finished, the intermediate results obtained to the entity pertinent in each case—depending once again on the communication pattern used by the framework—for the progressive construction of the final output.

In practice, beyond the mere deviations from the baseline referring to the point in time at which the stages pointed out occur, the communication architecture employed largely conditions both how the different tasks addressed are performed, and the roles or responsibilities of the nodes that make up the underlying hardware infrastructure. Specifically, when analyzing and comparing the communication behavior of the frameworks studied, three distinct mainstream communication patterns for dealing with node synchronization can be observed: (i) centralized, (ii) pipelined, and (iii) decentralized.

- The centralized architecture [70,71,72,78,80,81,84,86] stands out as the most implemented communication pattern for in-cluster co-inference. A MapReduce-like distributed programming model is used to provide a synchronized coordination of CNN inference computations across a given number of mobile and embedded devices. It has been found that using a distributed architecture such as MapReduce makes parallel data processing easier during DNN execution [76]. Its effectiveness has been proven in many ML applications on mobile platforms [77,78], maximizing the usage of computing resources of the nodes in a computing cluster [77]. To facilitate parallel processing, the Map procedure breaks down a job into smaller, more manageable chunks that can be executed in parallel, while the Reduce procedure brings together the data collected during the Map procedure’s intermediate stages.
  Regarding infrastructure, the model is implemented over a distributed network cluster formed by a central node, commonly referred to as the master node in the studies analyzed, but also as the group owner, gateway device, host device, and group leader, and multiple supplementary worker nodes, also known as slave devices, assisting devices, and follower nodes. A single device takes on the role of the central node and is responsible for coordinating the partitioning [80] and co-inference [72,86], which, according to the frameworks analyzed, may entail a broad spectrum of tasks: analyzing, splitting, and distributing input data [70]; registering participating devices and setting up the communications with them [86]; and managing the mechanisms underneath the data structures utilized for node coordination [73] such as IP address tables [72]. Worker nodes assist the master device by performing some of the computations required. During inference, each IoT device produces its own partial results, which are then aggregated to produce the final output of the system. Each worker node is mapped with only some of the partitions, which are processed and then reduced back to the central node, thus generating the input for the next layer considered in the Map procedure.
- The pipelined architecture [77,79,85,91] conceives the workflow as a sequence of n computation stages—corresponding to the n nodes in the hardware infrastructure—and n-1 communication steps for transferring intermediate results between adjacent devices [77]. Both the computation nodes and the execution flow are typically predetermined at configuration time, thereby simplifying task assignment into a mere sequential ordering, circumscribing the pursued objectives to find the split points that optimize the performance of the exploited CNN deployed. In this regard, whereas in [85], for example, each stage refers to the processing of a group of DNN layers on a subcluster, i.e., a subset of the devices constituting the overall system, in [79], partitioning is reduced to its minimal expression on a setup consisting of two devices, requiring a decision as to which portion of the DNN model should be executed locally and which should be offloaded to a second paired device. Finally, as far as node roles are concerned, generally speaking, and contrary to centralized architectures, there is not such an explicit distinction except for the fact that the first stage typically receives the input data, and the final stage is the one that generates the inference result [85].
- The decentralized architecture [74,89], as its name suggests, represents an even larger stepped divergence from the centralized communication pattern. In a centralized framework, as described above, worker nodes commonly need to register only with the central node, and the communication between them is typically bidirectional. Although bidirectional communication remains pertinent for the decentralized pattern, the latter requires much communication between each pair of nodes, thereby dramatically increasing the number of communications almost two-fold. Moreover, as there is no central node coordinating the other devices, each one must register with every other device, making that management far more complicated [74]. As made explicit in [89], each node holds information about how to coordinate itself with the rest of the computing entities in compliance with the intricate DNN layer dependencies, and, accordingly, each one is ultimately responsible not only for calculating the corresponding intermediate results, but also for forwarding them to the next intended device.

## 4. DNN Partitioning and Parallelism for Collaborative Inference

The analysis of the different mechanisms and techniques conceived to be an integral part of the software infrastructure underlying collaborative inference frameworks reveals a solid commitment to generating pertinent co-inference schemes towards enabling distributed DNN inference while adhering to the computational and economic profile of edge devices. In this context, partitioning and parallelism stand out as core elements of the collaborative inference paradigm in IoT systems, being the focus of interest and, thus, the primary direction of the related research efforts. Supported by the data collected in Table 3 and Table 4, we flesh out the analysis carried out throughout the previous section by further elaborating on the different parallelism and partitioning strategies exploited, as well as the different algorithmic solutions devised in the studies examined for implementing such strategies, thereby providing the reader with a comprehensive background of the practicalities of the built-in decision-making approaches in the co-inference frameworks, including those approaches that were specifically conceived for the various challenges that have arisen along those lines.

### 4.1. Taxonomy of Parallelism Strategies and Partitioning Schemes

Generally speaking, we identify in the body of literature surveyed two distinct yet complementary ways of parallelizing DNN tasks: applying the same DNN task on multiple inputs., i.e., data parallelism, and splitting down and distributing among multiple devices the computations required, i.e., pipeline parallelism and model parallelism. Thus, while data parallelism relies on the presence of multiple data inputs to parallelize computations, accomplishments and progress made on pipeline parallelism and model parallelism have been closely related to DNN architecture, the CNN architecture in particular. Their inner structure serves as a facilitating element for DNN partitioning and parallelism, making it an unavoidable point of analysis and an unquestionable reference in the effort to parallelize the involved calculus and successfully adjust the granularity to the capabilities and resources of the supporting infrastructure for an eventual improvement in model execution performance. 

Data parallelism is a straightforward method that allows independent data to be processed concurrently by duplicating devices that perform the same task or, in other words, that utilize the same DNN model. This could facilitate the reusability of data and increase the inference throughput [75]—the number of inferences per unit of time—nearly two-fold in the best-case scenario. Nonetheless, as evidenced by its single occurrence in the corpus analyzed [72]—shown in the “Parallelism” column in Table 3—implementing and applying such a parallelism scheme may not be effective for devices with limited resources or scenarios involving IoT devices because it does not change the computation and memory footprint per node [75,76] and it lacks the flexibility required to adjust the amount of computation on each node [76].

The two other DNN-structure-based parallelism strategies, pipeline parallelism and model parallelism, may supplement data parallelism—as reported in [72]—by dividing the DNN task of interest (instead of the input data) in each case, and then distributing the resulting subtasks to the various computing entities participating in the inference process. More specifically, and depending on the arrangement of the layers within the DNN model employed, the distributed DNN workload can be addressed either sequentially, i.e., pipeline parallelism, by atomically assigning the layers of the model to the different devices for computation, or in parallel, i.e., model parallelism, by processing layers concurrently to fully exploit the computing and memory resources of such devices.

Backed, in terms of the communication, by the pipelined architecture introduced in the previous section, pipeline parallelism [77,79,82,85,88,90,91] constitutes the simplest way to distribute the inference workload. It is a parallelism modality inherent to the traditional chain-like architecture of DNNs, which typically consists of a sequence of layers in which each layer’s output is dependent on the output provided by its previous layers. Similar to MCC and MEC solutions, the layer is assumed to be a DNN minimum operator, and it is consequently adopted as the partitioning unit for realizing a type of granularity that results in multiple groups or blocks of layers that can be spread across multiple compute nodes, reducing the burden on each of them and even increasing the end-to-end throughput. That being said, this layer-wise partitioning does not enable actual parallel processing of the partitions due to the noted data dependency between layers, also raising, as a consequence, significant design challenges. In this regard, adopting such granularity does not only make it possible for DNN partitions to be concurrently processed, but it also makes the memory footprint and complexity stemming from the partitions intractable for resource-constrained devices, in most cases.

Model parallelism [70,71,72,73,74,75,76,78,80,81,83,84,86,87,89] attempts to address both issues by employing partitioning strategies with a finer granularity to produce less expensive DNN subtasks, i.e., partitions with fewer parameters and fewer computation requirements than a layer, and foster more adaptable co-inference schemes. The computations required for a single input are distributed across multiple computing entities, reducing the time needed to process the shared input [76] but delivering a performance that, as opposed to what has been indicated for pipeline parallelism, is highly dependent on the distribution of such computations across devices. Table 3 provides valuable insights regarding model parallelism, clearly linking it to intra-layer partitioning. Only two research efforts [75,78] propose an approach halfway between model parallelism and pipeline parallelism, in the sense that, like pipeline parallelism, the approach relies on inter-layer partitioning, generating, in this case, branches that only communicate for input and pre-final activation, and can therefore be executed simultaneously on different devices, as model parallelism advocates.

Intra-layer partitioning techniques break down computations within layers while preserving dependencies according to DNN layer-specific aspects at the micro-architecture level. More specifically, intra-layer partitioning and the resulting model parallelism rely on the independence of the calculus within each layer. In that sense, their proper exploitation requires a careful understanding of DNN layer performance characteristics, prior knowledge of how DNN-task computations are performed, and an in-depth examination of the potential impact of both approaches on the computations and data communication among the computing units involved. Through a thorough analysis of the papers cited as a reference for this second partitioning and offloading approach, it is possible to provide a greater level of detail on the types of layers considered, how the various conceived partitioning schemes operate on them, and how these affect the parallelism ultimately achieved.

Although a CNN model consists of different kinds of layers, such as fully connected layers (also known as dense layers), convolutional layers, pooling layers, and activation functions, research on model parallelism (see Table 3 “Partitioning” column) has been primarily focused on conceiving intra-layer partitioning techniques and methods on convolutional and dense layers, resulting in novel vertical partitioning strategies of diverse natures and dedicated to different interests. This has been mainly motivated by the very nature and the subsequent memory and computing demands of the different layer types considered. Analyses carried out in this context [70,76,83] have confirmed that, while convolutional and fully connected layers account for the majority of the memory footprint and computational complexity of CNN models, the effect of non-parametric layers, i.e., pooling layers and activation functions, in this regard and, therefore, their contribution to the overall computational cost of the network, can be considered negligible [71]. Thus, when partitioned, the non-parametric layers are typically grouped with their corresponding parent layer, i.e., the layer that generated their input, instead of receiving explicit treatment [76].

Regarding the fully connected layers, the partitioning methods found in the analyzed literature fall into two distinct categories: input-based partitioning and output-based partitioning. Dense layers exhibit a simple inner structure in which all the input neurons are connected to all the output neurons, i.e., all the activations in the preceding layer, and each connection represents a weight, which is a multiplication operation [83]. As each input and output neuron is associated with multiple connections, each output of the preceding layer must have data dependencies on every output of the succeeding layer, necessitating communication when distributing their computation [74]. As the calculus for each activation is independent of that required to calculate other activations, it is possible to parallelize the computations of a dense layer, splitting the memory footprint, i.e., the saved weights, and the number of multiplications accordingly [76].

Output-based partitioning parallelizes the generation of each activation by distributing the computation of each output across multiple target devices [75] while copying all input values to each [76]. Weights sharing the same output neurons are assigned to the same computing entity, whereas intermediate results generated by preceding layers are distributed across all devices [83]. Once a device receives the input data, it initiates the computation necessary to generate these intermediate results, which are then merged to enable the consumption of the subsequent layer. Using an accumulative operation, the resultant values are concatenated in the proper order [76]. Input-based partitioning, in turn, splits the input across devices, resulting in lower communication overhead compared to the output-based approach. Each node calculates activations only partially, computing merely those portions of the output that are dependent on the received input [75]. Part of the input is transmitted to each device, which holds the weights corresponding to the specific input partition to be processed. Exploiting those values, the node performs the necessary computations and merges the intermediate results obtained with those transmitted from the previous layer by accumulation. Finally, once all the computing entities have completed their processing, the results are merged by summation, adding all the corresponding partial sums, an extra gather operation not required for the output-based alternative.

Although fully connected layers account for larger parameter size, both the time demands and dynamic memory usage of convolutional layers vastly outweigh those of fully connected layers [71], making them, to a large extent, the key constraining elements when trying to efficiently deal with the inference of DNNs [85]. In this regard, despite being considered the common bottleneck for DNN execution [71], convolutional layers have proven to be more conducive to computational parallelism: the computation of each filter, which generates each channel comprising the output feature maps, is independent [72], while, at the same time, parts of the output of the convolutional layer only require a subset of the input [76]. Accordingly, such intra-layer independency of computations has been extensively explored and leveraged by the authors, leading to a good number of partitioning solutions (see Table 3), specifically pursuing parallelism in this type of layer. Notably, these partitioning strategies fall into two broad categories: spatial and channel-wise partitioning.

Spatial partitioning stands out as the central strategy to partition feature maps, splitting each feature map along the spatial dimensions, i.e., height and width, into smaller tiles to then distribute their processing across different computing entities [81,85]. As each output element is only linked to a small part of the input feature maps, each original convolutional layer can be split into multiple concurrent DNN subtasks with smaller input and output regions, thereby increasing parallelism and reducing the memory footprint. Despite keeping all the filters in the memory field [69], each device works only on a subset of the feature maps that come out of the DNN, generating multiple outputs, which are then joined together spatially to produce the final result [76]. 

Partitioning schemes conceived along this line [71] initially tried to maintain a structural symmetry inspired by Coates et al. [93] by splitting the inputs along the two-dimensional space in the form of 2D grids [71,73,81,84]. However, the communication overhead caused by the need to extend each region with elements overlapping adjacent areas to compute convolution at the edges, aggravated by the synchronization overhead caused by DNN model’s inter-layer data dependency and the subsequent need to process them layer by layer, prompted the emergence of new approaches in search of possible solutions to both challenges. As a result, studies such as [70,80,86,87,91] developed alternative one-dimensional partitioning methods aimed at reducing the number of neighbors, and thus the overlapped areas, by splitting the input along a single spatial dimension—typically the longer one [70,80,87]—in the form of strips. Furthermore, although still based on the same 2D grid-based partitioning, research specifically focused on the synchronization problem [73,84] opted to fuse multiple layers, dividing the original DNN into tiled stacks in which most of the computations are performed locally, i.e., within the fused block, and only communication of the input of the first layer and the output of the last layer is required between devices.

For its part, channel-wise partitioning splits feature maps along channels and distributes the resulting segments across different nodes. Broadly speaking, although such a process requires each node to exchange their partially accumulated output feature maps to produce the final ones, consequently leading to significant communication overhead, it entails the computation of only one subset of the convolution filters per computing entity, thereby producing a set of non-overlapping channels [76]. Motivated by this, Du et al. [74,83] proposed an inter-channel-partitioning-based inference approach that leverages group convolutions [94,95] and channel shuffle [96] to decouple the original DNN structure for more effective model parallelism. Contrary to spatial partitioning, also known as intra-channel splitting, inter-channel partitioning divides the various input channels and filters among the multiple devices employed, requiring each of them to retain only the kernels of the allocated channel group, thereby facilitating an evenly distributed memory footprint. Moreover, since each filter only works on the feature maps of one group, there is no data dependence between channel groups. The output feature maps, the input feature maps, and the filters within the same group are entirely independent of other groups, thus leading to fewer synchronization points.

### 4.2. Decision Making for Partitioning Scheme Generation

The search for an optimal partitioning scheme delivering the best performance possible, subject to the requirements and characteristics of the DNN exploited, as well as the capabilities of the hardware infrastructure used for deployment, has gone beyond the mere interest in determining the type of parallelism strategy or the partitioning modality to be adopted. In general, it has emerged as a complex decision-making exercise in which the difficulty has stemmed primarily from the broad spectrum of factors involved and the considerable number of variables to be determined. Ultimately, a partitioning scheme must reflect the currently available network- and device-specific resources. Furthermore, due to the large number of computing units and layers with different complexities that typically comprise DNNs, partitioning and distributing such deep architectures may lead to a great variety of possible decompositions.

To maximize the performance of the available computing entities, the search for a suitable partitioning scheme has been modeled as an optimization problem, in which the exploration of the search space, according to the data collected in Table 3, is governed by quantitative measurements of the DNN model’s performance when executed across the system’s multiple nodes. More specifically, the objective of horizontally distributing a DNN to parallelize the computations required for inference is to improve the performance of the entire system, mainly in terms of time-specific metrics. Essentially it seeks to minimize latency [70,71,75,78,79,80,81,82,87,88,89,90] or maximize throughput, i.e., the number of performed inferences per second, when dealing with data streams [72,76,77,85], and, to a lesser extent and in terms of energy efficiency, to minimize power consumption in a few studies [79,85,86]. In particular, as regards the objective of minimizing latency, in nearly all the studies analyzed, this refers to the time cost incurred for the end-to-end execution of the exploited DNN model, being computed as the sum of the computation cost derived from the execution of the DNN partitions in the different devices involved and the transmission cost reflecting the time necessary to communicate intermediate results between nodes. Only a few exceptions deviate from the above: (i) considering only data delivery time as the time cost [70]; (ii) introducing certain nuances to the definition, e.g., Xu et al. [79] and Dhuheir et al. [82], which quantify latency as the sum of per-layer estimated execution time and the time elapsed from capturing an image until making the decision, respectively; or (iii) adopting a different time-related metric as an object, e.g., Zhang et al. [87], which uses the estimated gap between the least and most considerable time costs obtained from the computing entities.

Moreover, the complexity and, accordingly, the time required to search for the optimum, especially in those cases where optimization is modeled as an NP-hard problem [82,85,86,89,90], have been further exacerbated due to the urge to reconcile the noted performance objectives with problem-specific constraints, laying down user-defined requirements that limit the possible range of values the various objective factors and the variables considered for their calculation may take, delimiting in that manner the potential feasible regions where the final solution can be found, and ultimately resulting in a more restricted search space. In lieu of a more traditional classification based on the mathematical relationship represented in each case by the constraints, e.g., linear, non-linear, convex, it is possible to classify the constraints listed in Table 3 based on their semantics, identifying three distinct groups: performance constraints, infrastructure constraints, and policy constraints.

Performance constraints refer to restrictions on objective factors, such as the minimum end-to-end latency allowed [72,86], established to ensure their value is higher or, at least, not lower than a given user-defined threshold. On the other hand, infrastructure constraints are driven by the capabilities and state of the supporting hardware infrastructure, specifically the memory budget of the devices utilized [72,75,76,82,90], i.e., the maximum memory usage allowed, their computing power [70,71,75,82,90], which defines the maximum computational load tolerated, and their storage capacity [81]. Lastly, transformation policy constraints, as their name suggests, serve as directives that govern (i) how DNN layers are processed, for instance, forcing each layer to be computed by only one device [82,90] and avoiding overlapping [89]; and (ii) how the costs are calculated, indicating, for example, whether there is a transmission of intermediate results [82] or if a partition is processed by a particular device [82,90].

Over and above the specificities highlighted, objectives and constraints are used to define the partitioning problem formally, this being a key step towards a better understanding of the problem itself and, as a result, an essential task when it comes to developing optimal or, at the very least, adequate solutions. In this sense, the model constitutes a simplified abstraction of the problem, consisting of mathematical expressions that establish the relationship at play between the various factors considered, thus formalizing the potential outcomes of a given policy or scheme. Specifically, by attending to the data collected in this respect in Table 3, it is possible to further categorize the optimization problems observed in the literature surveyed into four distinct groups or subcategories: (i) workload balancing; (ii) direct acyclic graph (DAG) splitting; (iii) scheduling; and (iv) a last type encompassing classic problems in mathematical optimization.

Workload balancing (i) [70,71,72,76,77,78,80,87,88] in particular has emerged as the most prevalent problem model in the corpus under study. The relationship between performance and the balance of computation among devices is so strong that a hierarchy partition is deemed balanced if the ratio of processing times on the most and least loaded devices is minimized. In a distributed system, load balancing can optimize response time and even achieve real-time inference by allocating partitions to nodes in such a way that the amount of computation per node is nearly identical, thereby preventing the uneven overloading of some computing entities while others are idle. DAG splitting [79] (ii) constitutes a network optimization problem, which, based on graph theory, models the distributed inference process as a direct acyclic graph. The graph, laid out according to the configuration of the DNN being operated, represents the execution flow underlying the standard DL inference, delineating the existing potential execution paths. Finally, while job scheduling (iii) is deemed to be a variant of the traditional parallel machine job scheduling [81], targeting task–device mappings to enable the parallel processing of some given identical tasks on several processors without additional overhead [85], under the umbrella of classic mathematical optimization (iv), we find more straightforward equation-based formalizations in the form of integer linear programming [82,86] and non-linear programming [89,90] problems.

The nature of the problem addressed dictates the type of solution to be adopted in each case. The model abstracts the problem, providing a framework for either developing a brand-new algorithm from scratch or, more commonly, reusing and fine-tuning a well-known algorithm to rationalize the search for potential solutions. All the research efforts identified can be situated within this last line of work, essentially exploring and exploiting general problem-solving techniques, namely, (i) greedy algorithms [71,78,80,81,82,85,89], which recursively build up the pursued global optimum by picking the best partial solution at each iteration, for instance, partitions with the least computational complexity [71] or with the smallest total prediction time consumption [78], and also devices that accomplish the best latency with the maximum residual computation [82] or produce the slightest increase of the maximum task completion time [81]; (ii) exhaustive search algorithms [72,75,88], which sequentially evaluate each potential solution in order to eventually obtain the global final solution; (iii) heuristic algorithms [76,79], which lay down rules to allow a more efficient exploration of the search space, e.g., grouping partitions with the same latency [76] and pruning the DNN’s computationally light nodes [79], yielding a faster solution at the cost of sacrificing optimality or accuracy; and (iv) classic optimization methods [77,86,89], such as dynamic programming [77] and linear programming [86,89], which leverage mathematical models to solve problems directly, deriving solutions by optimizing, i.e., maximizing or minimizing, depending on the case, the objective function of interest while satisfying the set of constraints considered, and, in the particular case of dynamic programming, even simplifying the decision making by breaking it down into a sequence of decision steps over time.

### 4.3. Major Challenges and Specific Strategies Explored

The complexity of decision making, inter-layer dependency, and data redundancy resulting from the distributed processing of DNN partitions have subtly emerged in the two preceding subsections as typical obstacles the authors must overcome when designing partitioning strategies to maximize the parallelism of DNN tasks, necessitating the development of specific approaches. Both the complexity of the partitioning problem itself and the communication-specific matters noted above may hinder the performance objective pursued by the vast majority of the studies surveyed, as shown in Section 3. NP-hard problems, in particular, are inadequate for the online allocation of resources and distribution of tasks, whereas the synchronization and need for cross-partition data overlapping, both typical in parallel processing of DNNs with convolutional layers, directly impact, as mentioned in Section 4.1, the communication overhead between the interconnected IoT devices used, leading to frequent communications and large data transmissions, thereby resulting in higher end-to-end inference time.

Moreover, the disparity between these two problems becomes even more pronounced in terms of proposed solutions. Aside from the specificities of each particular strategy employed, there is a considerable difference between the two regarding the level of attention received and the variety of approaches developed to address them. Solving NP-hard problems (e.g., linear programming [86,89] and non-linear programming [90] problems) entails only the reformulation of the problem itself, either by adopting heuristics [82] or relaxing the assumed constraints (e.g., replacing discrete integer variables with continuous ones [74,86], or adding new decision variables to the formulation to convert a non-convex problem into a convex one [90]) to derive approximations allowing the desired partitioning schemes to be obtained more efficiently in the first instance, and ultimately real-time end-to-end processing.

In contrast, communication overhead arises in the studied corpus as the primary focus of interest among authors as far as the optimization required to achieve such performance levels is concerned, resulting in a greater variety of methods, as evidenced by the relatively important number of references collected in Table 4 in this regard. In addition to the strategies outlined in Section 4.1 for the design of more complex partitioning schemes—specifically, layer fusion [73,80,84] and inter-channel partitioning [74,83]—these methods include orthogonal optimization techniques, which are either oriented towards the improvement in operational aspects of DNN distribution and collaborative inference, in particular, scheduling strategies [73,74,77,83,87], such as the partitioning schemes just referred to, or oriented to the tailoring or adjustment of the exploited DNNs themselves, namely, DNN structure tailoring [71,74,75,77,83,84] and compression methods [81,91].

The work by Zhao et al. from 2020 [74] stands out as the only exponent concerning scheduling-related improvements in the related literature, reporting specific solutions to the transmission overhead and the computational redundancy problems resulting from spatial partitioning, particularly from the need to replicate overlapping input data between adjacent partitions in the various computational entities involved to produce fragments. To address both issues, the authors proposed an optimized scheduling process seeking to foster the reuse of the overlapped data, exploiting a caching mechanism to ultimately pursue a reduction in the amount of data transmitted. The implemented runtime achieves such a reduction by caching the overlapped intermediate feature map data produced when processing a partition, immediately making the data available to other neighboring partitions and rendering the data necessary only for the first layer of each partition to receive and generate the non-overlapping regions of input and intermediate feature maps. However, despite this improvement in both computational and data transmission performance, the reuse scheme resulted in dependencies between partitions that may hinder parallelism, thus prompting the design of additional scheduling mechanisms to parallelize work while taking such a dependence into account. To that end, overlapped intermediate data management was centralized on a gateway for efficiency, while the other nodes were programmed to process incoming requests in dependency order—more specifically, beginning with those with the smallest amount of overlap—thus maintaining the synchronization overhead low.

DNN structure tailoring refers to strategies that tweak factors or aspects regarding the inner structure of the DNNs to obtain architectures that are more conducive to efficiently running inherently tightly coupled models on distributed resource-constrained devices, thereby not only pursuing the minimization of communication costs but also accommodating, in the vast majority of cases, the size of the models of interest to the memory and computing resource budget of the devices comprising the exploited hardware infrastructure (see Table 4). In this respect, there is specific research [74,75,78,83] that has already emerged, as presented in Section 4.1, approaching the transmission overhead challenge by replacing standard convolutions with group convolutions and channel shuffle operations [74,83], and transforming a chain-like DNN model into several virtually independent and narrower branches by removing non-branch connections [75,77], leading in both cases to greater data independence between the processed units. Furthermore, on a slightly different track, we may include under the same umbrella techniques, typically employed at design time, that seek to achieve more efficient data transmission through communication compression: pruning [71,84], which exploits DNNs’ sparsity to simplify the structure of a given network by systematically removing redundant connections, for instance, zeroing out shared parts of the tensors [71], during training, resulting in a considerable decrease in transmission data size; and the use of encoder–decoder structures [77], which involve modifying the DNN’s original architecture at potential inter-device communication points by embedding a pair of symmetric networks, i.e., an encoder and a decoder, that acts as a bottleneck, compressing data to be transmitted at the origin and decompressing it at the destination once received.

These methods involve modifying the structure of the original DNN model by either reducing the number of connections [71,75,84] or using more efficient operators [74,83] in their layers. However, as indicated in the previous paragraph, they have a benefit beyond the reduction in communication overhead, decreasing the number of parameters and MAC operations and, consequently, the memory and computational demands, in general [71,74,75,83], which can affect, in turn, the quality of the predictions provided, causing accuracy loss. In order to mitigate this degradation, the prevailing trend in the works referring to this particular issue has been to complement or support the optimization mechanisms with strategies that enable recovering part of the lost accuracy. Specifically, as shown in Table 4, though the simple re-training of the network has proven to be an effective solution in studies experimenting with the sparsity of the model structure [71,81], in those involving adjustments at the architectural level, the accuracy recovery has also been implemented by means of solutions of this nature [74,75,83], increasing the number of channels and output features in convolution and fully connected layers [75], and applying channel shuffling operations after group convolutions to allow the exchange of information between groups [74,83].

In any case, despite the loss of accuracy noted, additional pure compression methods were also developed in a similar manner, aiming to also derive lighter DNN models, but unlike DNN structure tailoring techniques, this development was achieved by reducing the volume or numerical precision of the parameters comprising a trained DNN. Particularly in this line, the reviewed literature revealed two distinct approaches: quantization and more traditional compression methods. Data quantization reduces the number of bits used to represent the parameters, thereby lowering the size and computational complexity of the DNN models. In particular, Zhang et al. employ in [81] a scalar quantization approach, converting non-zero output values represented by 32-bit floating point numbers into lower-precision integer values. Moreover, when it comes to non-DNN-specific compression methods, the research of Zhang et al. [81] emerges again in the analysis, supplementing quantization with the application of a classic compression method, namely, run-length encoding [97], on intermediate results in an effort to reduce transmission costs even further. In this regard, it is important to note that, while the pursued goal has definitely been to reduce the memory size of the data exchanged between nodes, there has also been an evident concern for avoiding or, at the very least, mitigating information loss in the compression–decompression process, something that we see in [81] with the use of run-length encoding, and also in [91], where Parthasarathy et al. apply the LZ4 algorithm [98] for the same purpose, where both of them are lossless data compression methods.

On a different note, while still in the same context of study, flexibility has emerged as the other major challenge when developing intelligent IoT systems. Such systems consist of a number of nodes that might change in time [72,78]. As mentioned at the beginning of the document, these nodes are devices that do not necessarily have the same computational capabilities nor the same memory resources [71,78,79,85,86] that are typically scattered in the wild, where their communication capacity is affected by the changing conditions of the medium [77,78,79,86], but in some cases, such as unmanned aerial vehicles, have the ability to move around, which introduces new factors of variability such as the relative distance between devices [90], contributing to further dynamism. While in the reviewed papers it is possible to distinguish scheduling methods, such as the work stealing scheme [73,87] and the adaptive communication compression strategy [77], to adaptively adjust and distribute inference tasks to deal with the dynamic nature of workloads in IoT clusters, and while there are even studies that take device heterogeneity into account when formalizing the distribution problem, the main goal pursued along those lines has been to take a dynamic snapshot of the factors mentioned above that constitute the operational context [72,78,79,80,86], depicting the actual conditions prevailing at any given time to feed the system’s decision engine with the most reliable and up-to-date data possible and ultimately produce optimal action plans to cope with the DNN requests.

That said, the assessment of the system’s performance and the status of the production environment in real time, which are necessary for better responding to the fluctuation in available resources, may introduce an extra burden into the collaborative inference workflow, thereby affecting the system’s response time. Overall, such online monitoring is seen as an unsustainable solution to the search for an optimal DNN partitioning or distribution scheme in a timely manner, and, as such, most authors in the field have relegated accurate measurements of the system performance to a secondary role, relying instead on estimates or rough assessments based on the offline profiling of the devices and the DNN models concerned to feed the decision-making engine, thus enabling a more efficient co-inference generation. Profiling stands out, therefore, as a linchpin for streamlining the configuration and generation of co-inference plans. The approach encompasses techniques and methods that, overall, seek to characterize offline both the DNN tasks to be executed [72,78,79,87] and the infrastructure used to that end [79,81,86,90], resulting in models or data structures ready to be exploited later, online, to ultimately make the decision making feasible at runtime.

In particular, DNN profiling is typically carried out on each computing entity by first abstracting the model concerned, e. g., layer types [72,78,87], layer parameters [79,87], and each layer’s input size [78], and then capturing the characteristics of the hardware exploited (e.g., the computing capability [79,86], the power parameters [79,86], and the network status [79]), to derive the models [72,79,80,83] or just the profiled data archives [78,81,86] leveraged a posteriori to directly obtain the performance statistics (e.g., memory usage [72], latency [78,79,80,87], energy consumption [79], throughput [81], and computing intensity [86]) of the system in production from the configuration of the DNN model executed. More specifically, according to the nature of the assets obtained as a result in each case, it is possible to categorize the various profiling techniques reported in the surveyed literature into three distinct groups: (i) application-specific profiling techniques [78,86], which produce per-DNN-model profiles by running each DNN of interest on every particular device or hardware configuration comprising the system; (ii) statistical modeling methods [72,79,87], which commonly create specific profiles for the different types of layers within the DNN model studied [72,79] in a one-time offline effort resulting in application-independent predictive models (e.g., regression models [72]) able to estimate the performance of a given layer merely from aspects of its configuration; and (iii) a hybrid approach of (i) and (ii), represented by a single work [83] that establishes a linear regression model for each type of device used.

## 5. Discussion

Partitioning DNN inference into multiple subtasks and allocating resources to address them constitutes the core at the operational level of the collaborative intelligence studied throughout the present work, necessitating the identification of optimal values for the factors required to perform such partitioning and distribution. In this sense, the architecture of the DNN models used (predominantly chain-like), the type of DNN layers considered (primarily convolutional and fully connected layers), the structural characteristics of these layers, and the granularity of the partitioning emerge as fundamental aspects, governing both the DNN partitioning and the subsequent offloading and execution of the partitions produced, thereby dictating their number and complexity in terms of both memory and computation.

Intra-layer granularity has been embraced almost universally in the frameworks analyzed, with spatial partitioning adopted as the primary partitioning modality employed in the examined study context, resulting in notable commonalities between the reviewed studies in terms of both the partitions produced and their processing. With regard to partitioning, the majority of the papers examined report the generation of partitions consisting of small tiles (i.e., fragments of the feature maps) constituting subtasks that still have some data dependency but can be addressed concurrently across the system’s nodes. As for the calculations required to process the partitions, these have been primarily articulated using a MapReduce-like distributed programming model, coordinating the participation of the various devices according to a scheme in which a single node is responsible for managing the partitioning and subsequent co-inference, typically acting as the scheduler of the remaining worker nodes and an aggregator of the partial results generated by the latter.

Overall, this model parallelism has not been shown to necessarily affect the internal connections of a DNN. Although it does reduce the computational and memory footprint per node, the single-chain dependency between successive layers limits the scope of parallelism within a single inference. In addition, the requirement to extend each region with elements that overlap adjacent regions in order to compute convolution at the edges, along with the phenomenon just mentioned, results in significant communication overhead. This communication overhead has emerged, if not as the primary challenge, at least as the issue that has received the most attention in the corpus studied, resulting in the development of a wide range of specific advancements not only pertaining to the partitioning process itself (e.g., layer fusion and inter-channel partitioning) or the execution of the produced partitions (e.g., overlapped data reuse), but also with regards to the representation (e.g., quantization) and inner structure of the DNNs (e.g., pruning, as well as the exploitation of group convolutions and encoder–decoder structures) to ultimately derive lighter networks or networks with architecture more conducive to partitioning.

Aside from the communication overhead, the changing nature of the environment in which IoT systems typically operate—in particular, bandwidth variability—and the potential hardware configuration heterogeneity of devices that are part of them, have emerged as significant obstacles in the pursuit of optimal performance in terms of inference time. The trade-off between decision-making efficiency and system adaptability has motivated a plethora of related research, resulting in a substantial body of strategies and mechanisms that, on the one hand, have attempted to deftly produce co-inference schemes to ultimately minimize the impact on inference at runtime, but, on the other hand, have striven to make these schemes adaptive to the current state of the different components or aspects of both the exploited co-inference frameworks and their operating environment; ideally, this is by capturing online the current state of the system, but, in the vast majority of cases, it is by exploiting predictive models or instead making use of pre-computed profiling data to alleviate the burden.

A significant number of publications also exhibit a degree of laxity in assessing the proposed solutions or even present certain omissions in the evaluation report, which somewhat undermines the robustness of the results. Furthermore, in this respect, regarding the equipment used in the experimentation, we can see experiments in which controlled hardware and software configurations are used for this purpose, far from the conditions of a real-world environment, emulating various wireless network configurations via the use of a wired connection with a bandwidth limited by software [74,80,83,84], and even functioning in [89,91] with a fixed bandwidth. The results presented in the text refer in some cases to relative measures of performance, such as the acceleration obtained by certain methods relative to other alternatives [76,89], giving the reader an indication of the performance of the approaches studied instead of a perfectly accurate picture of it.

Beyond these minor flaws, the design of the evaluation protocol presents more profound challenges that must be addressed in future research. Below we detail the three identified.

- Regarding the design of the experiment, there is considerable heterogeneity, not only in terms of the evaluation metrics considered or the hardware and software infrastructure used, but also, and most importantly, in terms of the DNN models adopted, as well as the competing schemes and the specific aspects of analysis considered when conducting an in-depth study of the proposed solutions or their comparative analysis with other existing options. In this sense, the development of a common evaluation benchmark, at least for the most representative frameworks, would contribute to the creation of a more solid or scientifically rigorous groundwork in this context of study, enabling a unified comparison of these frameworks and the eventual abandonment of the current model, which adopts the local execution of models on a single device as baseline [71,72,79,81,83,86,88,91] for comparison.
- In a significant number of studies, certain factors, the majority of which are closely related to the hardware configuration used in each case, are not adequately evaluated or analyzed. A third or less of the co-authored papers do not address issues such as accuracy [71,74,75,77,83] (fundamental in user-centric intelligent systems), memory footprint [71,73,74,81,87,89], and communication overhead [70,71,73,82,84,90,91] (prevalent as major challenges in the smart IoT research reviewed), or energy consumption [72,75,79,81,86,88,91] (not unique to IoT solutions, but particularly problematic in these contexts). Therefore, it becomes evident that the study of such factors should be generalized to the extent possible.
- Although execution time assessment is discussed in nearly all of the reviewed studies, with the exception of [77,89], the vast majority of them lack depth or level of detail. In general, the analysis conducted in this context focuses almost exclusively on the end-to-end inference latency, omitting its treatment in relation to aspects or tasks of great importance that have emerged throughout the document, with detail found in only very few works, such as data transmission [70,71], pre-communication data transformation [91], and decision making for generation or updating the partitioning scheme [90].

Setting aside evaluation issues, but still focusing on system performance, the lack of interest in factors such as energy consumption and the precision mentioned above in relation to assessment has also been reflected in the design and implementation of mechanisms and strategies for the appropriate distribution of DL tasks and optimization of the execution environment, which have, in essence, pursued the viability of these intelligent services against memory and computational limitations. Having said that, co-inference frameworks must provide adequate levels of accuracy to ensure operational robustness without penalizing quality of service, all while taking energy consumption into consideration in order to, for example, extend device usage time per battery charge. In this context, whereas incorporating these factors into decision making is clearly a promising avenue to pursue, it will require potentially significant efforts from the scientific community, not only for the conception of models that are representative of the new problem posed, but also for the design or tuning of algorithms capable of achieving an adequate compromise between the design objectives envisaged.

In any case, it will be necessary to continue to delve into strategies that contribute to getting the execution time of the co-inference process closer to the real-time objective. In this context, even better exploitation of the resources available in the mobile and embedded devices that can be commonly found today in this type of infrastructure constitutes a possible avenue for improvement towards further reducing the end-to-end latency derived from the inference of the deployed DNN models. Specifically, the optimal exploitation of modern SoCs in many of today’s IoT devices will allow making use of the emerging generation of chipsets and their processing units with ad hoc designs to accelerate AI, i.e., neural processing units or NPUs, so as to locally parallelize the execution of DNNs [99]. However, the coordination of this intra-device parallelism with the inter-device strategies studied, as well as the partitioning and the horizontal and vertical distribution of DNNs, are anticipated to be a major challenge that must be faced in order to reach such performance levels.

Finally, although the body of work considered in the survey covers a broad spectrum of solutions that have enabled solid and agile progress on deep learning processing in IoT contexts, as we pointed out in Section 3, they have generally maintained the focus on building the foundations of the paradigm and, at a practical level, on the progressive improvement in the corresponding techniques. Consequently, they have not only disregarded an entire strand of work more focused on the design of approaches oriented more to support the needs derived from use cases or application domains of potential interest, but they have also adopted conventions for certain aspects to delimit and make the problems faced more approachable, at least in the current early stages of the analyzed research. In this sense, future research should expand or refine the techniques and methods developed to date, conceiving new approaches or adapting existing ones in order to provide an effective response to the particular challenges of the production context (e.g., adaptability in response to the changing nature of the operating environment and robustness to adverse environmental conditions), or to support both factors of greater complexity that had hitherto been virtually ignored (for instance, considering more modern yet complex DNNs with DAG architectures [78,79,87,89,91]) and progressively more sophisticated features (e.g., data stream processing [72,77,79,86,87,88]), which are fundamental in IoT environments, instead of using a static input.

## 6. Conclusions

Driven by the current momentum of IoT and DL, this paper outlines the most recent relevant research conducted to enable the processing of advanced tasks based on highly complex state-of-the-art DNNs in current IoT environments via collaborative intelligence implemented due to the aggregate power of multiple entities present in the execution framework. The typical limitations of memory and computing capacity in the involved devices are circumvented by parallel processing of the employed models, with this parallelism implemented at a practical level by partitioning the models and assigning the resulting partitions to the available or most appropriate nodes at any given time. Thus, it is possible to make better use of the resources or capabilities of IoT devices, which are typically insufficient to handle DL tasks, and address the entire end-to-end inference process at the same level of computation on relatively close entities geographically, bringing data processing closer to its source or acquisition entity and, most importantly, reducing the cost of communication between nodes and, thus, the overall inference time.

In particular, the study provides an exhaustive analysis of the principal approaches designed along these lines, which have been conceived as comprehensive, collaborative in-cluster inference solutions or, as the title indicates, methods aimed at enabling the horizontally distributed execution of DNN models in IoT environments. The analysis has focused on the most pertinent aspects relating to both the partitioning schemes used and the parallelism paradigms explored, offering an organized and schematic discussion of the underlying workflows and associated communication patterns, as well as those aspects or features at both macro- and micro-architectural levels of the DNNs that have guided the design of such techniques. Supplementing this core, the document pinpoints and analyzes the primary challenges encountered at the design and operational levels, e.g., decision making for the generation of partitioning schemes and the adaptability of the resulting frameworks to the dynamic nature of the IoT systems, as well as the specific adjustments explored in response to them, also providing a final discussion of the more salient insights derived from the research carried out and outlining several directions for future work. In this sense, the work was conceived as a practical guide that aims to be more than a mere compendium or summary of techniques, and we hope it will contribute to stimulating fruitful discussions and inspiring new research ideas to push the field forward.

## Figures and Tables

**Table 1:** Summary of the review papers pertinent to this work that exist in the literature.

Work	Year	Domain	Focus	Scope
[12]	2019	EC ∩ AI	Methods for fast inference	LTWT DNN, MOD ADPT, INF ACC, DIST INF, INF PRIV, DIST TRAIN, APPS
[27]	2019	EC	Computation offloading	CORE, APP PART, TSK ALLOC, DIST TSK, APPS
[20]	2019	EC ∩ AI	Operational challenges	HW, APPS, MULTI TEN, SCHED, MOB, SCAL, PRV, AI LC
[11]	2019	EC ∩ AI	-	CORE, DIST TRAIN, METR, MOD PART, MOD ADPT, MOD SEL, CACHE
[28]	2019	EC ∩ AI	Specialized hardware	HW
[22]	2019	EC ∩ AI	-	CORE, DIST TRAIN, MOD PART, MOD ADPT, INF ACC
[25]	2019	ECC	Computation offloading	MULTI TEN, WKLD BAL, MOB, PART TYPES
[26]	2020	EC ∩ AI	Communication challenges	COMM CHLG, COMM-EFF TRAIN, COMM-EFF INF
[24]	2020	DIST ML	-	CORE, ML WF, TOPO, DIST FMW, DIST ML TRAIN
[18]	2020	EC ∩ AI	-	CORE, HW, DL FWK, DIST TRAIN, CACHE, MOD ADPT, MOD SEL, MOD PART, OFLD TYPES, APPS
[23]	2020	EC ∩ AI	-	DIST TRAIN, LTWT DNN, MOD ADPT, INF ACC, OFLD TYPES, APPS
[21]	2021	EC ∩ AI	-	DIST TRAIN, LTWT DNN, MOD ADPT, DIST INF, DL FWK, HW, APPS
[17]	2022	EC ∩ AI	Training techniques	DIST TRAIN, MOD PART, MOD ADPT, PRETRAIN, EDG PREPROC, BC, LTWT DNN, APPS
[19]	2022	EC ∩ AI	-	CORE, LTWT DNN, MOD ADPT, MOD PART
[29]	2022	ECC ∩ AI	ML-based analytics	DT ANAL, DIST TRAIN, MOD PART, PARAL, TSTB
[16]	2022	ECC ∩ AI	Architectures for collaborative learning	CORE, DIST TRAIN, INF OFLD, BI COLLAB, LTWT DNN, MOD ADPT, RL, APPS

Abbreviations: EC, edge computing; ECC, edge–cloud computing; EC ∩ AI, edge intelligence; ECC ∩ AI, intelligence through the edge–cloud continuum; DIST ML, distributed machine learning; LTWT DNN, lightweight deep neural networks; MOD ADPT, model adaptation; METR, performance metrics; MOD PART, model partitioning; MOD SEL, model selection; INF ACC, inference acceleration; DIST INF, distributed inference; INF PRIV, privacy preservation for inference; DIST TRAIN, distributed training; APPS, application scenarios; CORE, core concepts; APP PART, application partitioning; TSK ALLOC, task allocation; DIST TSK, distributed task execution; HW, specialized hardware; MULTI TEN, multi tenancy; SCHED, scheduling; MOB, mobility awareness; SCAL, Scalability; PRV, privacy and trust; AI LC, AI lifecycle; CACHE, edge caching; RL, reinforcement learning; DL FWK, DL frameworks; OFLD TYPES, offloading modalities; PART TYPES, partitioning modalities; WKLD BAL, workload balancing; TSTB, testbeds; PARAL, parallelism schemes; DT ANAL, data analytics; INF OFLD, inference offloading; BI COLLAB, bidirectional collaboration; COMM CHLG, communication challenges; COMM-EFF TRAIN, communication-efficient training; COMM-EFF INF, communication-efficient inference; ML WF, machine learning workflow; TOPO, cluster topologies; DIST ML TRAIN, distributed machine learning training; DIST FMW, distributed computing frameworks; BC, blockchain; EDG PREPROC, edge pre-processing; PRETRAIN, cloud pre-training.

**Table 2:** Recent research efforts that study the distribution of DL workloads within an IoT cluster.

						Architecture		Workflow	
Work	Year	Approach	Objectives	DL Tasks	Applications	Communication	Tier	Offline Setup	Runtime
MoDNN [70]	2017	FWK	Lower OVR LAT	IMG CLASS	GEN	Centralized	LAN	PART	CO-INF
MeDNN [71]	2017	FWK	Lower OVR LAT Lower TX OVHD	IMG CLASS	GEN	Centralized	LAN	PART TSK ASSG	CO-INF
Musical Chair [72]	2018	FWK	Real-time DNN CMPT	IMG CLASS AR	GEN	Centralized	LAN	PART TSK ASSG	TSK ASSG CO-INF
DeepThings [73]	2018	FWK	Lower MEM FP	OBJ DET	GEN	Centralized	Edge	PART TSK ASSG	TSK ASSG CO-INF
[74]	2020	FWK	High ACC	IMG CLASS	GEN	-	LAN	DNN TWK PART TSK ASSG	CO-INF
LCP [75]	2020	DNN SPT	Lower TX OVHD	IMG CLASS	GEN	Decentralized	Edge	-	-
[76]	2020	DNN DIST	Higher TPUT Lower OVR LAT	IMG CLASS VID CLASS	GEN	-	Edge	PART TASK ASSG	TSK ASSG
[77]	2020	FWK	Higher TPUT Lower OVR LAT	IMG CLASS IMG ST ANAL	GEN	Pipelined	Edge	PART TSK ASSG DNN TWK	DNN TWK CO-INF
[78]	2020	TSK ASSG	Lower OVR LAT	IMG CLASS	GEN	Centralized	Edge	TSK ASSG	TSK ASSG
DeepWear [79]	2020	FWK	No ACC loss	IMG CLASS AR DOC CLASS EMO RECOG SP RECOG	WEAR ANAL	Pipelined	LAN	-	PART TSK ASSG CO-INF
EdgeLD [80]	2020	FWK	Lower OVR LAT	IMG CLASS	GEN	Centralized	LAN	-	PART TSK ASSG CO-INF
ADCNN [81]	2020	FWK	ACC-LAT TO	IMG CLASS	GEN	Centralized	Edge	DNN TWK PART	TSK ASSG CO-INF
[82]	2021	TSK ASSG	Lower DM LAT	IMG CLASS	UAV SV	-	Edge	-	TSK ASSG
DeCNN [83]	2021	FWK	Lower OVR LAT	IMG CLASS	GEN	-	LAN	DNN TWK	PART TSK ASSG CO-INF
[84]	2021	FWK	Lower OVR LAT Lower TX OVHD	IMG CLASS OBJ DET	GEN	Centralized	Edge	DNN TWK	PART TSK ASSG CO-INF
PICO [85]	2021	FWK	Lower OVR LAT	IMG CLASS OBJ DET	GEN	Pipelined	LAN	-	PART TSK ASSG CO-INF
CoEdge [86]	2021	FWK	Lower OVR LAT Lower POW	IMG CLASS	GEN	Centralized	Edge	-	PART TSK ASSG CO-INF
DeepSlicing [87]	2021	FWK	Lower OVR LAT	IMG CLASS	GEN	-	Edge	USER-DEF PART	TSK ASSG CO-INF
[88]	2022	DNN DIST	Higher TPUT	-	GEN	-	Edge	-	-
EdgeFlow [89]	2022	FWK	Lower OVR LAT	IMG CLASS OBJ DET	GEN	Decentralized	Edge	PART	TSK ASSG CO-INF
[90]	2022	TSK ASSG	Lower OVR LAT No ACC loss	IMG CLASS	UAV SV	-	Edge	-	TSK ASSG
DEFER [91]	2022	FWK	Higher TPUT	IMG CLASS	GEN	Pipelined	Edge	-	PART TSK ASSG CO-INF

Abbreviations: FWK, framework; DNN SPT, DNN splitting; DNN DIST, DNN distribution; TSK ASSG, task assignment; OVR LAT, end-to-end inference latency; TX OVHD, communication overhead; DNN CMPT, DNN computations; MEM FP, memory footprint; ACC, accuracy; TPUT, inference throughput; ACC-LAT TO, accuracy-latency trade-off; DM LAT, decision-making latency; POW, energy consumption; IMG CLASS, image classification; AR, activity recognition; OBJ DET, object detection; VID CLASS, video classification; IMG ST ANAL, image data stream analysis; DOC CLASS, document classification; EMO RECOG, emotion recognition; SP RECOG, speech recognition; GEN, generic; WEAR ANAL, wearable device analysis; UAV SV, unmanned aerial vehicles surveillance; TIME CRIT, time-critical; LAN, local area network; PART, partitioning; CO-INF, co-inference; DNN TWK, DNN tweaking; USER-DEF PART, user-defined partition scheme.

**Table 3:** Overview of the more relevant aspects of parallelism and partitioning for collaborative inference across IoT devices.

			Decision-Making			
Work	Parallelism	Partitioning	Problem Model	Objectives	Constraints	Solution
[70]	Model PAR	1D input PART for CL	WKLD BAL	MIN LAT	DEV CMPT	-
[71]	Model PAR	2D grid input PART for CL	WKLD BAL	MIN LAT	DEV CMPT	Greedy algorithm
[72]	Data PAR Model PAR	Output-based PART for FL	WKLD BAL	MAX TPUT	DEV MEM SZ LAT	Exhaustive search
[73]	Model PAR	2D grid input PART for CL	-	-	-	-
[74]	Model PAR	Group-wise PART for CL Input-based PART for FL	WKLD BAL	-	-	-
[75]	Model PAR	Inter-branch PART	WKLD BAL	MIN LAT	DEV MEM SZ DEV CMPT	Exhaustive search
[76]	Model PAR	-	WKLD BAL	MAX TPUT	DEV MEM SZ	Heuristic algorithm
[77]	Pipeline PAR	Layer-wise PART	WKLD BAL	MAX TPUT	-	Dynamic programming
[78]	Model PAR	Inter-branch PART	WKLD BAL	MIN LAT	-	Greedy algorithm
[79]	Pipeline PAR	Layer-wise PART	DAG SPLIT	MIN POW MIN LAT	-	Heuristic algorithm
[80]	Model PAR	1D input PART for CL	WKLD BAL	MIN LAT	-	Greedy algorithm
[81]	Model PAR	2D grid input PART for CL	SCHED	MIN LAT	DEV STOR	Greedy algorithm
[82]	Pipeline PAR	Layer-wise PART	ILP	MIN LAT	MAX MEM MAX WKLD Layer per node Binary control	Greedy algorithm
[83]	Model PAR	Inter-channel PART for CL Input-based PART for FL	-	-	-	-
[84]	Model PAR	2D grid input PART for CL	-	-	-	-
[85]	Pipeline PAR	Layer-wise PART	SCHED	MAX TPUT	-	Dynamic programming Greedy algorithm
[86]	Model PAR	1D input PART	INLP	MIN POW	LAT	Primal simplex algorithm
[87]	Model PAR	1D input PART	WKLD BAL	MIN LAT	-	-
[88]	Pipelining	Layer-wise PART	WKLD BAL	MIN LAT	-	Exhaustive search Heuristic algorithm
[89]	Model PAR	1D input PART	ILP	MIN LAT	No overlapping	-
[90]	Pipeline PAR	Layer-wise PART	NLP	MIN LAT	DEV MEM SZ DEV CMPT	-
[91]	Pipeline PAR	Layer-wise PART	-	-	-	-

Abbreviations: PAR, parallelism; CL, convolutional layer; PART, partitioning; WKLD BAL, workload balancing; DAG SPLIT, direct cyclic graph splitting; SCHED, scheduling; ILP, integer linear programming; INLP, integer non-linear programming; NLP, non-linear programming; MIN LAT, minimizing inference latency; MAX TPUT, maximizing inference throughput; MIN POW, minimizing energy consumption; DEV MEM SZ, device memory size; LAT, inference latency; DEV CMPT, device computing capacity; DEV STOR, device storage capacity; MAX MEM, maximum memory usage; MAX WKLD, maximum tolerated computational load.

**Table 4:** Summary of the challenges and approaches conceived in the reviewed studies.

	Memory Footprint	Communication Overhead	Computational Burden	Inference Accuracy	Inference Efficiency	System Variability	Decision-Making
Scheduling strategies	[87]	[73,74,83]	[73]	-	-	[73,77,87]	-
Partitioning strategies	[80]	[73,74,80,81,83,84,86]	[81]	-	[84]	-	-
DNN structure tailoring	[75,83,84]	[71,74,75,77,83]	[71,75,83]	[74,75,83]	[71]	-	-
DNN retraining	-	-	-	[71,81]	-	-	-
Compression methods	-	[81,91]	-	-	-	-	-
Profiling	-	-	-	-	-	[72,78,79,80,81,86,87,90]	-
Problem reformulation	-	-	-	-	-	[71,85]	[82,86,89,90]

## Data Availability

Not applicable.
